# Using Current Smoking Prevalence to Project Lung Cancer Morbidity and Mortality in Georgia by 2020

**DOI:** 10.5888/pcd10.120271

**Published:** 2013-05-09

**Authors:** Victoria N. Davis, Antionette Lavender, Rana Bayakly, Kenneth Ray, Tamira Moon

**Affiliations:** Author Affiliations: Antionette Lavender, Rana Bayakly, Kenneth Ray, Tamira Moon, Georgia Department of Public Health, Atlanta, Georgia.

## Abstract

**Introduction:**

Tobacco use is the leading preventable cause of disease and premature death in the United States. In Georgia, approximately 18% of adults smoke cigarettes, and 87% of men’s lung cancer deaths and 70% of women’s lung cancer deaths are due to smoking. From 2004–2008, the age-adjusted lung cancer incidence rate in Georgia was 112.8 per 100,000 population, and the mortality rate was 88.2 per 100,000 population.

**Methods:**

The Georgia Behavioral Risk Factor Surveillance System Survey was used to estimate trends in current adult smoking prevalence (1985–2010). Georgia smoking–attributable cancer mortality was estimated using a method similar to the Centers for Disease Control and Prevention’s Smoking-Attributable Morbidity, Mortality, and Economic Costs application. Data on cancer incidence (1998–2008) were obtained from the Georgia Comprehensive Cancer Registry, and data on cancer deaths (1990–2007) were obtained from the Georgia Department of Public Health Vital Records Program.

**Results:**

From 1985 through 1993, the prevalence of smoking among Georgians declined by an average of 3% per year in men and 0.2% in women. From 2001 through 2008, lung cancer incidence rates declined in men and increased in women. Lung cancer mortality rates declined in men and women from 2000 through 2007. By 2020, Georgia lung cancer incidence rates are projected to decrease for men and increase for women. Lung cancer mortality is projected to decrease for both men and women.

**Conclusion:**

The lung cancer mortality rates projected in this study are far from meeting the Healthy People 2020 goal (46 per 100,000 population). Full implementation of comprehensive tobacco-use control programs would significantly reduce tobacco-use–related morbidity and mortality.

## Introduction

Tobacco use declined in the United States during recent decades, but smoking prevalence has remained unchanged during the past few years (1,2). The result is the occurrence of more than 400,000 premature and preventable deaths each year in the United States due to tobacco use (1,2). Each year approximately 10,300 adults aged 35 years or older in Georgia die prematurely from tobacco-use–related illnesses. These deaths represent approximately 1 of every 6 deaths among adult Georgians, making tobacco use a leading preventable cause of disease and premature mortality in the state. Smoking causes $1.8 billion in direct medical costs and $3.4 billion in lost productivity costs in Georgia each year (3).

The 1964 Surgeon General’s report, which described the relationship between smoking and lung cancer as causal, is among the most important reports related to cancer and tobacco use (4). Lung cancer is the most commonly diagnosed cancer and the leading cause of cancer death in the United States (2,5). During 2004–2008, the age-adjusted lung cancer incidence and mortality rates in Georgia were 112.8 and 88.2 per 100,000 population, respectively. Tobacco-use–related cancers (TRCs) are heavily researched and are used as an evaluation mechanism for tobacco-use and cancer control programs (5). TRCs refer to acute myelogenous leukemia (AML) and cancers of the oral cavity, pharynx, larynx, and esophagus; pancreas and stomach; kidney and renal pelvis; urinary bladder; lung and bronchus; and cervix (5).

In this article, we describe smoking prevalence from 1985 through 2010 among adults in Georgia and smoking-attributable mortality of TRCs and present trends in Georgia lung cancer incidence and mortality rates. The objective of this study was to use the current prevalence of adult cigarette smoking to project future incidence of lung cancer through 2020.

## Methods

### Current smoking prevalence

The current smoking prevalence among adults aged 18 years or older in Georgia was assessed using data from the Georgia Behavioral Risk Factor Surveillance System (BRFSS). Established by the Centers and Disease Control and Prevention (CDC) in 1984, the BRFSS is a cross-sectional random-digit–dialed telephone survey of noninstitutionalized civilian adults aged 18 years or older conducted annually in all 50 states, the District of Columbia, Puerto Rico, Guam, and the US Virgin Islands. This state-based surveillance system collects information on health-risk behaviors, preventive health practices, and health care access primarily related to chronic disease and injury. We used BRFSS survey data from 1985 through 2010 to calculate adult smoking prevalence and weighted the data to adjust for differences in probability of selection and nonresponse.

Smoking prevalence and 95% confidence intervals were calculated for adults overall and by sex and race/ethnicity. Statistically significant differences between proportions were determined using the Rao-Scott χ^2^ test, with significance set at *P* < .05 using SAS version 9.2 (SAS Institute, Inc, Cary, North Carolina). Survey respondents who said they had smoked 100 or more cigarettes in their lifetime and now smoke every day or on some days were defined as current smokers. Respondents who said they smoked 100 cigarettes or more in their lifetime and now do not smoke at all were defined as former smokers. The average annual percentage change in adult smoking prevalence for 1985 through 1993 and 2003 through 2010 were calculated by 1) subtracting the prevalence of the final year of interest from the prevalence of the initial year of interest and 2) dividing this difference by the prevalence of initial year of interest and multiplying by 100. This percentage change was then divided by the total number of years to determine the average annual percentage change.

### Smoking-attributable mortality

A smoking-attributable fraction method similar to the CDC’s Smoking-Attributable Morbidity, Mortality, and Economic Costs (SAMMEC) application was used to estimate the percentage of cancer deaths attributable to smoking during 2003 through 2007. SAMMEC derives smoking-attributable mortality (SAM) estimates using an attributable-fraction formula. The smoking-attributable fractions (SAFs) of adult deaths for 19 smoking-related diseases (including 10 TRCs) were calculated using age- and sex-specific smoking prevalence and relative risk (RR) of death for adult current and former smokers aged 35 or older. Age-adjusted RR estimates for adults aged 35 or older from the second wave of the American Cancer Society’s Cancer Prevention Study (CPS-II) 6-year follow-up were used (6). The percentage of current and former adult smokers aged 35 to 64 and aged 65 or older were obtained from the 2007 Georgia BRFSS to calculate the SAFs. SAFs for each disease and sex were derived by using the following formula:

SAF = [*p1*(*RR1* – 1) + *p2*(*RR2* – 1)] / [*p1*(*RR1* – 1) + *p2*(*RR2* – 1) + 1]

where

*p1* = Percentage of adult current smokers in study group

*p2* = Percentage of adult former smokers in study group

*RR1* = Relative risk of death for adult current smokers relative to adult never smokers

*RR2* = Relative risk of death for adult former smokers relative to adult never smokers

The age- and sex-specific adult SAFs were applied to adult mortality data for each smoking-related disease in the population being studied. Mortality data by cause of death in Georgia for 2003 through 2007 were obtained from the Georgia Vital Records system (7). The average annual number of deaths was multiplied by the relevant SAF for each smoking-related cause of death. The following formula was used to calculate the SAM:

SAM = Number of Deaths × SAF

Summing across 5-year age categories provided the sex-specific estimate of SAM for each cause of death. The average annual SAM is the sum of smoking-attributable deaths across age groups and across causes of death for both sexes combined.

### Lung cancer incidence and mortality

Cancer incidence cases (1998–2008) were obtained from the Georgia Comprehensive Cancer Registry (8), which is affiliated with CDC’s National Program of Cancer Registries. TRC definitions and codes were derived on the basis of a 2008 CDC publication (5). Cancer site and morphology codes were taken from the *International Classification of Diseases for Oncology, Third Edition* (ICD-O-3) (9). The codes for each site are as follows: lung and bronchus (C34), larynx (C32), oral cavity (C00–14), esophagus (C15), stomach (C16), pancreas (C25), kidney and renal pelvis (C64), urinary bladder (C67), cervix (C53), and AML (M9840, 9861, 9866, 9867, 9871–9874, 9891, 9895–9897, 9910, 9920) (5). Georgia incidence rates were calculated for 2004 through 2008.

Age-adjusted incidence rates were calculated per 100,000 population for adults aged 35 or older, using the direct method adjusted to the 2000 US standard population. Incidence rates were calculated with respect to the cancer-specific smoking-attribute risk percentage. Except where calculated to show trends, incidence rates reflect the average annual rate for the periods 1998–2001 and 2001–2008. Incidence rates were calculated for non-Hispanic whites and non-Hispanic blacks. However, for 1998 and1999, the black population includes the Hispanic population.

Cancer death data (1990–2007) were obtained from the Georgia Department of Public Health, Vital Records Program. TRC definitions and codes were based on the same codes described for incidence cases. Site codes were used based on the *International Classification of Diseases*, *Ninth* *and* *Tenth* *Revisions* (ICD-9 and ICD-10, respectively) and the Surveillance, Epidemiology, and End Results (SEER) Cause of Death Recode (10). Deaths occurring from 1990 through 1998 were categorized using ICD-9 codes, and deaths occurring from 1999 through 2007 were categorized using ICD-10 codes.

Age-adjusted mortality rates were calculated per 100,000 population for adults aged 35 or older using the direct method, adjusted to the 2000 US standard population. Mortality rates were calculated with respect to the cancer-specific smoking-attribute risk percentage. Except where calculated to show trends, rates reflect the average annual rate for the intervals 1990–1995, 1995–2000, and 2000–2007. Calculated mortality rates do not distinguish ethnicity.

Trend analysis by cancer site was performed for cancers with attributable risk percentages that are higher than 50% (oral cavity, larynx, esophagus, and lung and bronchus). Trends in 10-year age-specific incidence and mortality rates beginning with adults aged 35 to 44 years were analyzed for lung cancer. Annual percentage change (APC) was determined by 1) calculating the age-adjusted rate for each year, 2) subtracting the initial age-adjusted rate (AAR_o_) from the final age-adjusted rate (AAR_f_), 3) dividing by the initial age-adjusted rate, 4) dividing the answer by the number of years in the interval (n), and 5) multiplying by 100:

**Figure:**
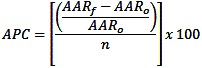


Projected lung cancer incidence and mortality rates were determined by 1) calculating the age-adjusted rates for each year; 2) multiplying the age-adjusted rate by the respective smoking-attributable rate for all adults, men, and women; and 3) multiplying the rate by the APC from 2001 to 2008 for incidence rates and 2000 to 2007 for mortality rates. We used SAS version 9.2 to generate age-adjusted incidence and mortality rates and age-specific incidence and mortality rates.

## Results

### Adult smoking prevalence

**Figure 1:**
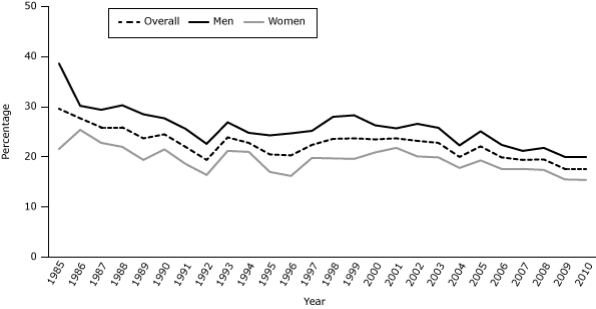
Current adult smoking prevalence, Georgia, 1985–2010. Current adult smoking is defined as adults aged 18 or older who have smoked at least 100 cigarettes in their lifetime and now smoke every day or on some days. Source: Georgia Behavioral Risk Factor Surveillance System. **Table:** 

The overall prevalence of cigarette smoking among adults declined from 29.6% in 1985 to 17.6% in 2010 (Figure 1). However, this has been a slow decline over the past 2 and a half decades. Increases in smoking prevalence were seen in 1993, 1997–2002, and 2005. However, since 2006, the overall adult smoking prevalence has remained stable (18%–20%). Smoking among men declined from 38.6% in 1985 to 20.0% in 2010. Smoking among women declined from 21.6% in 1985 to about 15.4% in 2010. From 1985–1993, the smoking prevalence among adult men in Georgia declined by an average of 3% per year, and it declined among women by an average of 0.2% per year. In Georgia, smoking has decreased by an average of 3% per year from 2003 through 2010 for adult men and women.

Year	Overall, %	Men, %	Women, %
1985	29.6	38.6	21.6
1986	27.7	30.2	25.4
1987	25.9	29.4	22.8
1988	25.9	30.3	22.0
1989	23.7	28.5	19.4
1990	24.5	27.7	21.5
1991	22.0	25.6	18.6
1992	19.4	22.6	16.4
1993	23.9	26.9	21.2
1994	22.8	24.8	21.0
1995	20.5	24.3	17.0
1996	20.3	24.7	16.2
1997	22.4	25.2	19.8
1998	23.6	28.0	19.7
1999	23.7	28.3	19.6
2000	23.5	26.3	20.9
2001	23.7	25.7	21.8
2002	23.2	26.6	20.1
2003	22.8	25.8	19.9
2004	20.0	22.3	17.8
2005	22.1	25.1	19.3
2006	19.9	22.4	17.6
2007	19.4	21.2	17.6
2008	19.5	21.8	17.4
2009	17.6	20.0	15.5
2010	17.6	20.0	15.4

Table 1 shows a comparison of the smoking prevalence in 2003 (n = 7,651) with the current smoking prevalence in 2010 (n = 5,788) by race/ethnicity and sex. Smoking prevalence has decreased significantly since 2003 for men and women overall. However, when categorized by race/ethnicity and sex, significant declines were observed for only non-Hispanic white men and women and Hispanic women.

**Table 1:** Current Adult Smoking Prevalence^a^, by Race/Ethnicity and Sex, Georgia, 2003 and 2010

Sex and Race/Ethnicity	2003, % (95% CI) (n = 7,651)	2010, % (95% CI) (n = 5,788)	*P* Value^b^
**Overall**	22.8 (21.4–24.1)	17.6 (16.0–19.2)	<.001
**Men**	25.8 (23.5–28.1)	20.0 (17.4–22.9)	.002
Non-Hispanic white	25.6 (23.0–28.3)	19.5 (16.5–22.9)	.006
Non-Hispanic black	25.7 (20.4–31.0)	22.2 (17.1–28.3)	.38
Hispanic	18.2 (8.6–27.8)	25.0 (10.0–49.9)	.39
**Women**	19.9 (18.3–21.4)	15.4 (13.8–17.1)	<.001
Non-Hispanic white	21.7 (19.8–23.5)	17.5 (15.5–19.7)	.004
Non-Hispanic black	14.9 (12.0–17.8)	12.1 (9.3–15.7)	.22
Hispanic	18.7 (8.8–28.6)	7.2 (3.5–14.2)	.03

Abbreviation: CI, confidence interval.

a Current adult smoking is defined as adults aged 18 years or older who have smoked at least 100 cigarettes in their lifetime and now smoke every day or some days.

b Rao-Scott χ^2^ test, significance set at *P* < .05.

### Smoking-attributable mortality

In Georgia men in 2007, 26.6% aged 35 to 64 years and 57.6% aged 65 or older were former smokers; 21.5% aged 35 to 64 years and 11.8% aged 65 or older were current smokers. Approximately 20.6% of Georgia women aged 35 to 64 years and 30.8% aged 65 or older were former smokers, and 18.7% aged 35 to 64 years and 8.5% aged 65 or older were current smokers. Approximately 16% of all deaths among adults aged 35 or older in Georgia resulted from cigarette smoking (total number of all deaths for this age group during 2003–2007 was 62,186). Of these deaths, smoking caused 43% of all deaths from cancer. The major cause of cancer death was tracheal, lung, or bronchial cancer; 87% of these cancer deaths among men and 70% of these cancer deaths among women were attributed to smoking (Table 2).

**Table 2:** Smoking-Attributable Cancer Mortality^a^, Adults Aged 35 or Older, by Sex, Georgia, 2003–2007

Cancer type	% Attributable to Smoking, Women	% Attributable to Smoking, Men
Trachea, lung, bronchus	70	87
Larynx	72	82
Lip, oral cavity, pharynx	45	73
Esophagus	57	71
Urinary bladder	28	46
Kidney, other urinary	5	37
Stomach	12	27
Acute myeloid leukemia	11	22
Pancreas	23	21
Cervix, uterine	11	NA

Abbreviation: NA, not applicable.

a Smoking-attributable mortality is the estimated percentage of cancer deaths attributed to cigarette smoking.

### Lung and bronchus cancer incidence and mortality rates

During 2004–2008, the incidence rate of lung and bronchus cancer was higher among men (165.1 per 100,000 population) than women (74.5 per 100,000 population). Black men (172.4) had the highest incidence rate when compared with white men (165.0), white women (80.1), and black women (56.9). Overall, lung cancer incidence rates increased by 2% per year from 1998–2001 and decreased by 1.1% per year from 2001–2008 (Table 3). Incidence rates among women increased by 3.7% per year from 1998–2001 and 0.4% from 2001–2008. Incidence rates for men increased from 1998–2001 followed by a decrease of 2.2% per year from 2001–2008. The largest increase occurred among white women (3.8%) from 1998–2001, and the largest decrease occurred in black men (3.0%) from 2001–2008.

**Table 3:** Annual Percentage Change (APC) in Age-Adjusted Incidence and Mortality Rates, Lung Cancer, Adults Aged 35 or Older, by Sex and Race, Georgia

Rate	APC for Men			APC for Women		
	Total	Black	White	Total	Black	White
Age-adjusted incidence						
1998–2001	0.8	0.5	1.0	3.7	2.8	3.8
2001–2008	−2.2	−3.0	−2.0	0.4	0.7	0.6
Mortality						
1990–1995	−0.5	−1.3	−0.2	2.5	2.2	2.5
1995–2000	−2.2	−3.4	−1.9	1.8	3.2	1.5
2000–2007	−2.2	−2.1	−2.3	−0.8	0.5	−0.8

During 2003–2007, the mortality rate for lung cancer was nearly 3 times higher among men (137.2 per 100,000 population) than women (53.9). Black men (145.6) had the highest mortality rate when compared with white men (137.8), white women (57.8), and black women (43.3). White women (57.8) had a higher mortality rate than black women (43.3). Overall, mortality rates increased slightly from 1990–1995 by 0.4% per year, followed by a decrease of 0.7% per year from 1995–2000 and a decrease of 1.6% from 2000–2007 (Table 3). The largest decrease occurred among black men (3.4%) from 1995–2000, and the largest increase occurred among black women (3.2%) during that same period. Among men, mortality rates decreased during all 3 periods. Among women, rates increased during 1990–1995 and 1995–2000, and a slight decrease was observed during 2000–2007.

### Lung cancer incidence and mortality projections

**Figure 2:**
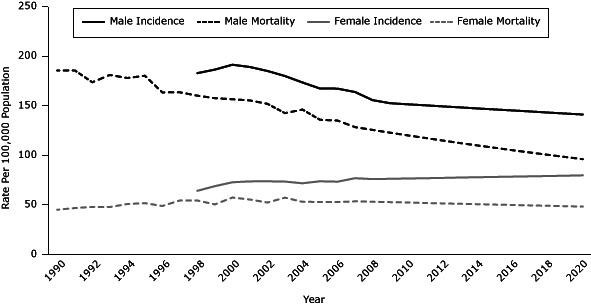
Age-adjusted lung cancer incidence rates (1998–2008), mortality rates (1990–2007), incidence rate projections (2009–2020), and mortality rate projections (2008–2020), adults aged 35 years or older, by sex, Georgia. Incidence data were not available for men and women from 1990 through 1997. Average annual rates per 100,000 population were age-adjusted to the 2000 US standard population. Projections were calculated by multiplying the age-adjusted rate by the respective smoking-attributable rate for all adults, men, and women. The rates calculated were then multiplied by the annual percentage change from 2001 through 2008 for incidence rates and 2000 through 2007 for mortality rates. Source: Georgia Comprehensive Cancer Registry. **Table:** 

 In 2020, lung cancer incidence rates for Georgia are projected to be 96.5 per 100,000 population for all adults aged 35 or older, 141.1 for men, and 79.8 for women (Figure 2). Among men, this figure represents an overall decrease of 9.0% from 2008 through 2020 or an annual decrease of 1.0%. The overall increase among women is projected to be 5.0%, which corresponds to a 0.4% increase per year.

Year	Men		Women	
	Incidence	Mortality	Incidence	Mortality
1990	—	185.5	—	45.1
1991	—	185.6	—	46.8
1992	—	173.6	—	48.0
1993	—	181.0	—	47.8
1994	—	177.9	—	50.9
1995	—	180.2	—	51.8
1996	—	163.4	—	48.9
1997	—	163.5	—	54.5
1998	182.8	160.1	64.2	54.4
1999	186.4	157.6	68.9	50.5
2000	191.3	156.5	72.8	57.4
2001	189.0	155.3	73.7	55.4
2002	185.0	151.9	73.8	52.4
2003	179.9	142.5	73.5	57.3
2004	173.4	146.1	71.8	53.1
2005	167.3	135.8	73.8	53.0
2006	167.2	135.0	73.4	52.8
2007	163.8	128.4	76.9	53.6
2008	155.6	125.6	76.0	53.2
2009	152.5	122.8	76.3	52.8
2010	151.4	120.1	76.6	52.4
2011	150.4	117.5	76.9	51.9
2012	149.3	114.9	77.2	51.5
2013	148.3	112.3	77.6	51.1
2014	147.2	109.9	77.9	50.7
2015	146.2	107.5	78.2	50.3
2016	145.2	105.1	78.5	49.9
2017	144.2	102.8	78.8	49.5
2018	143.1	100.5	79.1	49.1
2019	142.1	98.3	79.4	48.7
2020	141.1	96.1	79.8	48.3

In 2020, lung cancer mortality rates for Georgia are projected to be 68.6 per 100,000 population for all adults aged 35 or older, 96.1 for men, and 48.3 for women (Figure 2). Among men, this figure represents an overall decrease of 25% from 2007 through 2020 or an annual decrease of 2.0%. The overall decrease among women is projected to be 10.0%, which corresponds to an annual 1% decrease.

## Discussion

Lung cancer incidence and mortality rates projected in this study were assumed to be attributable to the smoking prevalence occurring 15 to 35 years prior (during 1985–1993). The exposure period of smoking to lung cancer morbidity and mortality can range from 5 to 40 years (11–13). The risk of lung cancer drops by as much as half even 10 years after a smoker quits (14). The smoking prevalence among men and women has declined significantly in Georgia during the past decade. Because lung cancer rates can begin to decline as soon as 5 years after smoking rates decline, lung cancer incidence rates are expected to decline in Georgia (2). However, since 2006, the overall smoking prevalence in Georgia has remained stable at 18% to 20%. Current smoking among men in Georgia declined by an average of 3% per year during 1985 through 1993, and smoking among women declined by only 0.2% during the same period. If smoking prevalence remains at the current rate, lung cancer rates will follow the current pattern through year 2020, according to our projections. We projected that by year 2020 these percentage changes in smoking prevalence will lead to a decrease in lung cancer incidence among men and an increase among women. By 2020, the lung cancer mortality rate among men in Georgia is predicted to be 96.1 per 100,000 population and 48.3 among women, projections that are far from meeting the Healthy People 2020 goal of 46 per 100,000 population.

During the 12-year period of the Georgia Tobacco Use Prevention Program’s existence, several major efforts may have contributed to some of the increases and decreases and eventual stabilization of the current smoking prevalence in Georgia. Some of these programming efforts have included media campaigns, counseling through the tobacco quitline, and free nicotine replacement therapy. CDC has recommended and supported state implementation and sustaining of comprehensive tobacco-use control programs to discourage smoking initiation, encourage smoking cessation, and protect nonsmokers from secondhand smoke (2,15).

Also recommended are tobacco-free policies that target elementary and high schools, colleges and universities, and parks and recreation facilities (14,15). Georgia has been able to target major programming efforts in only certain regions of the state, which may cause smoking prevalence to remain stable and prevent lung cancer morbidity and mortality rates from decreasing significantly (2,15,16). Full implementation of the state’s tobacco-use control program would sustain and intensify efforts to reduce cigarette smoking and secondhand smoke exposure; increase state excise taxes; implement model smoke-free air ordinances; enforce restrictions on tobacco advertising, promotion, and sponsorship; increase statewide mass media campaigns; and provide access to effective cessation interventions (15,16). These efforts would save billions of dollars being spent on medical and lost productivity costs (16).

Our study has limitations. The prevalence of smoking was determined using self-reported data from the BRFSS, which may be influenced by recall bias. Because a landline survey was used, adults with wireless-only service were not included. These adults are known to smoke more than the rest of the population, so smoking prevalence provided may have been underestimated (2). However, data were weighted to allow survey findings to be generalizable to Georgia’s population. The attributable-fraction methods used to calculate smoking-attributable mortality uses the smoking prevalence and number of deaths for the current year of data available. However, most deaths attributed to smoking result from smoking in previous decades, during which the smoking prevalence was higher. Therefore, the percentage of smoking-attributable deaths presented may be underestimated (6). Information pertaining to smoking status is not contained in cancer registry data. Therefore, the direct causal association between smoking status and the development of cancers presented cannot be determined. Also, other risk factors may affect cancer incidence and mortality rates, which may also play a role in the lung cancer projections. However, only the percentage of cancers that are attributable to smoking were applied to the projected rates, which may help control for other possible risk factors. The Georgia data used for cancer incidence rates is gold certified by the North American Association of Central Cancer Registries as high quality. An evaluation of the Georgia Comprehensive Cancer Registry showed that 100% of cancer cases are true cases.

## References

[1] Centers for Disease Control and Prevention. Vital signs: current cigarette smoking adults aged ≥18 years — United States, 2005–2010. MMWR Morb Mortal Wkly Rep 2011;60(35):1207–12. 21900875

[2] Centers for Disease Control and Prevention. State-specific trends in lung cancer incidence and smoking — United States, 1999–2008. MMWR Surveill Summ 2011;60(36):1243–7. 21918494

[3] Georgia Department of Public Health. Georgia adult tobacco use summary; 2012. http://health.state.ga.us/pdfs/epi/cdiee/2011/2011AdultTobaccoUse2.pdf. Accessed March 22, 2012.

[4] Doll R , Hill AB . Lung cancer and other causes of death in relation to smoking: a second report on the mortality of British doctors. BMJ 1956;2(5001):1071–81. 10.1136/bmj.2.5001.1071 13364389 PMC2035864

[5] Centers for Disease Control and Prevention. Surveillance for cancers associated with tobacco use — United States, 1999–2004. MMWR Surveill Summ 2008;57(SS-8). 18772853

[6] Centers for Disease Control and Prevention. Smoking-attributable mortality, morbidity, and economic costs (SAMMEC): adult SAMMEC and maternal and child health (MCH) SAMMEC application; 2002. https://apps.nccd.cdc.gov/sammec/methodology.asp.

[7] Georgia Vital Records. Atlanta (GA): Georgia Department of Public Health; 2008.

[8] Georgia Comprehensive Cancer Registry. Atlanta (GA): Georgia Department of Public Health; 2009.

[9] Fritz A , Percy C , Jack A , Shanmugaratnam K , Sobin L , Parking D , International classification of disease for oncology. Geneva (CH): World Health Organization; 2000.

[10] National Cancer Institute. SEER cause of death recode 1969+. Surveillance Epidemiology and End Results; 2010. http://seer.cancer.gov/codrecode/1969+_d09172004/index.html. Accessed December 20, 2010.

[11] Heloma A , Nurminen M , Reijula K , Rantanen J . Smoking prevalence, smoking-related lung diseases, and National Tobacco Control legislation. Chest 2004;126(6):1825–31. 10.1378/chest.126.6.1825 15596680

[12] Jemal A , Cokkinides V , Shafey O , Thun M . Lung cancer trends in young adult: an early indicator of progress in tobacco control (United States). Cancer Causes Control 2003;14(6):579–85. 10.1023/A:1024891201329 12948289

[13] Bach P , Kattan M , Thornquist M , Kris M , Tate R , Barnett M , Variations in lung cancer risk among smokers. J Natl Cancer Inst 2003;95(6):470–8. 10.1093/jnci/95.6.470 12644540

[14] US Department of Health and Human Services. The health consequences of involuntary exposure to tobacco smoke: a report of the surgeon General. Atlanta (GA): US Department of Health and Human Services, Centers for Disease Control and Prevention, National Center for Chronic Disease Prevention and Health Promotion, Office on Smoking and Health; 2006.

[15] Centers for Disease Control and Prevention. Best practices for comprehensive tobacco control programs — 2007. Atlanta (GA): US Department of Health and Human Services, Centers for Disease Control and Prevention, National Center for Chronic Disease Prevention and Health Promotion, Office on Smoking and Health; October 2007.

[16] Centers for Disease Control and Prevention. Vital signs: current cigarette smoking adults aged ≥18 years — United States, 2005–2010. MMWR Morb Mortal Wkly Rep 2011;60(35):1207–12. 21900875

